# Surgical Management and Outcomes of Pediatric Congenital Head and Neck Teratomas: A Scoping Review

**DOI:** 10.1002/oto2.66

**Published:** 2023-08-09

**Authors:** Siddharth Patel, Ansley J. Kunnath, Jean‐Nicolas Gallant, Ryan H. Belcher

**Affiliations:** ^1^ Vanderbilt University Medical Scholars Program Nashville Tennessee USA; ^2^ Meharry Medical College Nashville Tennessee USA; ^3^ School of Medicine Vanderbilt University Nashville Tennessee USA; ^4^ Department of Otolaryngology–Head & Neck Surgery Vanderbilt University Medical Center Nashville Tennessee USA

**Keywords:** congenital teratoma, cervical teratoma, head and neck

## Abstract

**Objective:**

To perform a scoping review to characterize postoperative outcomes of pediatric patients (ages 0‐18) with a history of congenital head and neck teratomas.

**Data Sources:**

PubMed, EMBASE, Web of Science, Cochrane, Clinicaltrails.gov.

**Review Methods:**

A search of multiple databases was performed. Studies were included if they detailed the surgical management and outcomes of pediatric patients with a history of congenital head and neck teratomas.

**Results:**

One hundred and eight studies totaling 137 patients were identified. The median gestational age at birth was 37 weeks. Respiratory distress, prompting emergent endotracheal intubation or tracheostomy, was present in most patients (58%). The ex utero intrapartum treatment (EXIT) procedure was utilized for 21 (15%) patients. The teratomas were resected after a median duration of 4 days from birth. The most common postsurgical complications were vocal cord paralysis (3%), hemorrhage (2%), and tracheomalacia (2%). Death occurred perioperatively in 2 patients (2%). Twenty‐six patients (19%) required additional surgery, and 5 patients (4%) needed adjuvant chemotherapy. Patients were monitored for a median duration of 24 months with a recurrence rate of 6%. Four recurrent cases (50%) had intracranial extension, and 88% of the recurrent cases were mature teratomas at initial histopathological diagnosis.

**Conclusion:**

Most patients with congenital head and neck teratomas require emergent airway management perinatally. Excisional and surgical complications are rare, and most patients are cured of their disease with a single operation. Recurrent teratomas tend to have an intracranial extension and are likely to be of mature pathology at the time of initial diagnosis.

Teratomas are germ cell tumors composed of ectoderm, mesoderm, and endoderm.^1^, ^2^ Common locations for these tumors include the sacrococcygeal region, reproductive organs, anterior mediastinum, and retroperitoneum.^2^ Teratomas are most often asymptomatic and are commonly found incidentally in adulthood; however, these masses can be found in neonates. Such congenital teratomas make up 3% to 5% of all teratomas, with an incidence ranging from 1 in 20,000 to 40,000 live births.^3^ Of these congenital teratomas, 6% arise in the head and neck region.^3^

Congenital head and neck teratomas that are found in the soft tissues of the face, upper aerodigestive tract or neck of neonates, can pose a significant risk due to compressive effects on the airway. Infants with these tumors can be born with fatal respiratory distress due to mass effect or related pulmonary hypoplasia, especially if not identified during the prenatal period.^3^ Sometimes an ex utero intrapartum treatment (EXIT) procedure—in which the partially delivered infant is kept on the placental blood supply while airway management takes place—needs to be performed due to the presence of these masses.^4^, ^5^ If endotracheal tube placement or tracheostomy fails during EXIT, an operation on placental support (OOPS) can be performed to resect the mass.^6^, ^7^

Although most congenital head and neck teratomas are benign, about 5% are malignant on final pathology—compared to 10% for congenital teratomas in all anatomic locations.^8^, ^9^ Similar to other tumors, histologic evaluation is necessary to determine the malignant potential of teratomas. The histology of a teratoma is classified into 2 major categories: mature and immature. Primitive neuroepithelium or neuroglial cells are primarily present in immature teratomas. The maturity of the teratoma does not correlate with malignant potential.^3^ Malignancy is thought to be dependent on the finding of foci of malignant germ cell tumors such as choriocarcinoma or yolk sac tumor.^3^ Possible sites of metastasis include cervical lymph nodes, lungs, mediastinum, and liver.^10^ Management of malignant teratomas is similar to other malignant germ cell tumors. Postsurgical surveillance includes repeated alpha‐fetoprotein (AFP) measurements and imaging. Continued elevation in AFP levels can indicate metastasis or recurrence.^11^ Adjuvant chemoradiotherapy and further surgical resection are mainstays for these metastatic tumors.^12^

Literature regarding the surgical management of congenital head and neck teratoma is limited. Early surgical resection with clear margins is deemed effective for long‐term disease‐free survival for patients with teratoma.^3^ For unknown reasons, however, recurrent teratomas have been reported in the literature.^13^ There are 2 systematic reviews addressing etiology, pathology, diagnosis, and treatment of these tumors.^6^, ^7^

Neither of these discuss postoperative outcomes, additional surgeries needed, or recurrence rates. Our objective is to conduct a literature review to further our understanding of postoperative outcomes and modalities of tumor surveillance during follow‐up visits. Due to the rarity of literature on congenital head and neck teratoma cases, a scoping review would provide an ideal methodology to include all types of studies, including gray literature.

## Methods and Material

### Eligibility Criteria

All articles detailing patients who (1) were born with a teratoma in the head and neck region and (2) underwent neonatal excision were initially considered. Those with orbital or intracranial teratomas were excluded. Since our primary objective was to study postoperative outcomes, we only selected patients who survived until the teratoma excision surgery. Moreover, articles without postoperative data were also excluded.

### Information Sources

PubMed, EMBASE, Web of Science, Clinicaltrials.gov, and Cochrane databases were queried. All potential types of studies were eligible for inclusion, including gray literature. Due to the retrospective nature of data collection from existing literature, this study was exempted from the patient consent requirement after the IRB assessment.

### Search Terminology

A search strategy was developed with the assistance of a research librarian. This strategy relied on MeSH (Medical Subject Headings) terms and was customized for each of the information sources. For PubMed, the search terminology was (“Teratoma”[Mesh] OR teratoma[tiab] OR teratomas[tiab]) AND (“Head and Neck Neoplasms”[Mesh] OR “Head”[Mesh] OR “Neck”[Mesh] OR “Oral”[Mesh] OR “Nasal”[Mesh] OR “Oropharyngeal”[Mesh] OR “Laryngeal”[Mesh] OR head[tiab] OR neck[tiab] OR cervical[tiab] OR oral[tiab] OR nasal[tiab] OR oropharyngeal[tiab] OR laryngeal[tiab]) AND (“congenital” [Subheading] OR congenital[tiab]). Appendix A presents search terminology for all databases utilized. No date limits were imposed. The last search was performed on 12/2/2021.

### Selection of Articles

**Figure 1:**
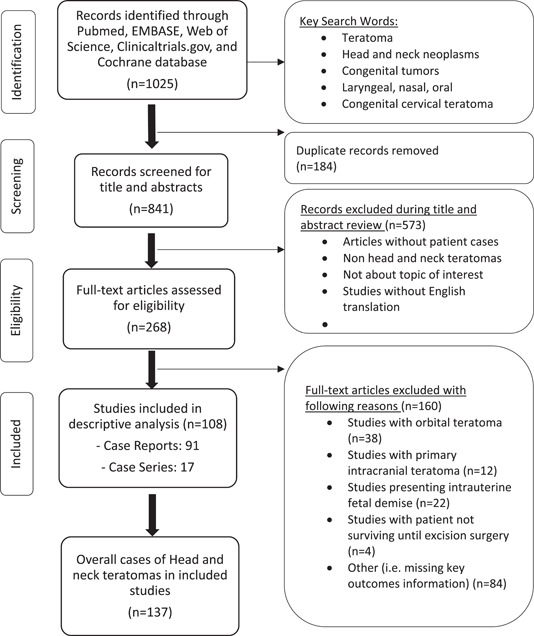
Flow diagram of evidence collection and screening (Figure summarizes the data curation process, highlights reasons for study exclusion, and description of included studies).

The initial search from PubMed, EMBASE, Web of Science, Clinicaltrials.gov, and Cochrane yielded 1025 articles. Duplicate records were identified using a reference manager (Endnote, Clarivate) and removed. The remaining articles were transferred to a literature review screening tool (Rayyan Professional, Rayyan Systems) for further review. Two independent reviewers (S.P. and A.K.) conducted title and abstract screening to identify 268 articles aligned with inclusion criteria. We excluded any studies without patient data, teratomas in other anatomic locations, and studies without English translation. Conflicts were resolved by an independent third reviewer (J.G. or R.B.). A full‐text article review was performed by the same 2 independent reviewers (S.P. and A.K.) on 268 articles identified previously (Figure 1). Conflicts during full‐text article reviews were also resolved by the same third independent reviewer (J.G. or R.B.). All reviewed articles were either case reports or case series. We categorized articles discussing single patient experience as “case reports” and multiple patients as “case series.”

### Data Abstraction

We developed a database (REDCap, Vanderbilt University) to collect variables of interest.^14^, ^15^ A list of data items was synthesized in collaboration with all key study personnel. S.P. conducted data extraction, and A.K. reviewed the extracted data for accuracy. Conflicts were resolved by J.G. or R.B.

### Synthesis of Results, Statistics, and Reporting

Data collected in REDCap were analyzed with SPSS (IBM) and Excel (Microsoft). Normality was tested using Shapiro‐Wilk tests. This study followed the Preferred Reporting Items for Systematic reviews and Meta‐Analyses extension for Scoping Reviews (PRISMA‐ScR) Guidelines.^16^

## Results

### Data Characteristics

A total of 108 eligible articles were identified. Of these, 91 articles were case reports, and 17 were case series. Appendix B presents a brief description of articles utilized for data extraction.

### Patient Characteristics

The literature search identified 137 patients with congenital cervical teratomas (Appendix B). Most patients were white (n = 81/137, 59%) or Asian (n = 25/137, 18%); a minority were black, Native American, or other. The mean maternal age at birth was 27.7 ± 5.2 years. The most reported maternal prenatal complication was polyhydramnios (n = 26). Most pregnancies reached term (n = 95/137, 69%), with 38 (n = 38/137, 27%) pregnancies resulting in preterm delivery. Eighteen of the preterm births were associated with polyhydramnios prenatally. The median gestational age at delivery was 37 weeks.

### Perinatal Airway Management

At delivery, 80 patients had respiratory distress requiring intervention (n = 80/137, 58%). Patients with severe respiratory distress typically required endotracheal intubation (n = 63/137, 46%). EXIT was successfully utilized to intubate 21 patients (n = 21/137, 15%). One patient successfully underwent tracheostomy during their EXIT procedure. All patients who were intubated without requiring tracheostomy at delivery (with or without EXIT) were weaned off of respiratory support postoperatively after a median duration of 13 days and a range of 2 to 270 days (**Table** 1).

**Table 1:** Summary of Patient Demographics and Clinical Presentation

	Patients (n = 137)	Percentage (%)
Sex		
Female	75	55
Male	49	36
Unknown	13	10
Race		
Caucasian	81	59
Black	7	5
Asian	25	18
Native American	5	4
Native Hawaiian	0	0
Unknown	19	14
Ethnicity		
Hispanic	6	4
Non‐Hispanic	131	96
Maternal age in years (mean ± SD)	27.7 ± 5.2	
Gestational age		
Term	95	69
Preterm	38	28
Unknown	4	3
Median (weeks)	37	
Presurgical diagnostic imaging		
CT	59	43
MRI	51	37
X‐ray	7	5
Ultrasound	7	5
Unknown	13	10
Maternal prenatal and L&D complications		
Polyhydramnios	26	19
Pre‐eclampsia	1	1
Hemorrhage	1	1
Infection	1	1
Preterm labor	2	2
None documented	106	77
Respiratory distress		
Yes	80	58
No	57	42
Airway management		
Supplemental oxygen	15	11
Endotracheal intubation	63	46
Nasopharyngeal airway	2	2
None documented	57	42
EXIT		
Yes	21	15
No	59	43
None documented	57	42
Intubation duration		
Range	2‐270	
Median	13.5	

### Surgical Details

Computed tomography (CT) (n = 59/137, 43%) and magnetic resonance imaging (MRI) (n = 51/137, 37%) were the mainstay of imaging to understand the extent of the disease preoperatively. Eleven (n = 11/137, 8%) patients had intracranial extension and 10 (n = 10/137, 7%) patients had mediastinal extension. Patients underwent surgical intervention with a median duration of 4 days from birth and a range of 0 to 330 days. We noted low intraoperative and postoperative complications. Three (n = 3/137, 2%) patients suffered from intraoperative hemorrhage requiring blood transfusion, and 1 patient had unilateral internal carotid artery injury. The most commonly reported postoperative complications were vocal cord paralysis (n = 4/137, 3%), tracheomalacia (n = 2/137, 2%), and wound infection (n = 2/137, 2%). One patient was also noted to have hypothyroidism due to excision of thyroid gland with the specimen (n = 1/137, 1%). Reported postoperative death occurred in 2 patients in this study (n = 2/137, 2%). Due to the complex nature of these teratomas, 26 (n = 26/137, 19%) patients required additional surgeries. Out of these patients, 22 only required 1 additional surgery, whereas 3 patients required 2 surgeries and 1 patient required 3 surgeries to ensure complete removal of the tumor. A tracheostomy was required in 20 patients at the time of tumor resection, in addition to 1 patient at birth. They were all decannulated after a median duration of 60 days. Patients possibly undergo direct laryngoscopy prior to decannulation, however, these procedures were not detailed in the studies reported. Four (n = 4/137, 3%) patients required gastrostomy tube placement. Five (n = 5/137, 4%) patients were treated with adjuvant chemotherapy (Table 2). Of these 5 patients receiving adjuvant Chemotherapy, 3 patients had histologic evidence of malignancy. The other 2 were immature teratomas.

**Table 2:** Surgical Variables and Complications

Variables	Proportions (n = 137)	Percentage
Presurgical diagnostic imaging		
CT	59	43%
MRI	51	37%
X‐ray	7	5%
Ultrasound	7	5%
Unknown	13	10%
Weight (kg) at time of surgery		
Mean ± SD	2.7 ± 0.8	
Birth to surgery duration, days		
Range	0‐330	
Median	4	
Intracranial extension	11	8%
Mediastinal extension	10	7%
Intra‐op complications		
Hemorrhage	3	2%
Vascular injury	1	1%
Postop complications		
Hypothyroidism	1	1%
Tracheomalacia	2	2%
Vocal cord paralysis	4	3%
Wound infection	2	2%
Aspiration pneumonia	1	1%
Post‐op death	2	2%
Patients requiring additional surgeries	26	19%
Number of additional surgeries		
1	22	16%
2	3	2%
3	1	1%
Adjuvant chemotherapy	5	4%
Reported G‐tube placement	4	3%
Duration or g‐tube, days		
Median	45	
Reported tracheostomy	21	15%
Duration of tracheostomy, days		
Median	60	

### Tumor Details and Outcomes

Congenital head and neck teratomas were most commonly located in the oral cavity or oropharynx (n = 44/137, 32%) and anterior to the sternocleidomastoid in the neck (n = 40/137, 29%). Tumor volume varied greatly, with a range of 10 to 2925 cc and a median of 72 cc. On the final histopathology report, the majority of the teratomas had mature components (n = 104/137, 76%), and 33 (n = 33/137, 24%) had immature neural tissue.

Patients were followed up for 1 to 134 months with a median duration of 24 months. Most of the patients were followed up with either CT (n = 41/137, 30%) or MRI (n = 49/137, 36%). Eight (n = 8/137, 6%) patients had a tumor recurrence. Recurrence duration from surgery was 3 months to 5 years. Three of these patients were asymptomatic, and the recurrence was identified on follow‐up imaging. Two patients presented with new nasal obstruction, and subsequent physical exam identified the recurrent tumors. Elevated AFP was noted in the remaining 1 patient, and imaging confirmed the suspected recurrence. All patients underwent revision surgery, ensuring clear margins. One patient received neoadjuvant chemotherapy to reduce the tumor burden. One patient received adjuvant chemotherapy due to distal metastasis identified on imaging. Seven of these patients did not have any further recurrence on subsequent follow‐ups. The 1 patient with metastatic disease continued to have new tumor foci at distal sites despite adjuvant chemotherapy (Tables 3 and 4).

**Table 3:** Tumor Details and Outcomes

Variables	Proportion (n = 137)	Percentage (%)
Location of mass		
Oropharyngeal	44	32
Nasopharyngeal	24	18
Lingual	11	8
Anterior cervical	40	29
Posterior cervical	3	2
Thyroid	6	4
Other	9	7
Tumor volume cc (n = 93)		
Median	72.0	
Range	2‐2925	
Histology		
Mature	104	76
Immature	33	24
Maximum follow‐up duration (months)		
Range	1‐134	
Mean	41.6	
Median	24	
Recurrence	8	6
Follow‐up modality		
CT	41	30
MRI	49	36
U/S	0	0
Physical exam	37	27
None mentioned	10	7

**Table 4:** Characteristics of Recurrent Teratomas and Follow‐up Detection Modality

Cases	Initial diagnosis	Original site	Location of recurrence	Modality of detection	Time of detection
1	Mature	Oropharyngeal	Infratemporal fossa	MRI	3 months
2	Immature	Anterior cervical	Metastasis to lungs	AFP	4 months
3	Mature	Nasopharyngeal	Infratemporal fossa	CT	5 months
4	Mature	Oropharyngeal	Nasal cavity	Physical exam	5 months
5	Mature	Anterior cervical	Infratemporal fossa	CT	7 months
6	Mature	Oropharyngeal	Oropharyngeal	Physical exam	9 months
7	Mature	Nasopharyngeal	Nasopharyngeal	Physical exam	2 years
8	Mature	Anterior cervical	Infratemporal fossa	Physical exam	5 years

## Discussion

This study sought to characterize surgical management and related outcomes for patients born with head and neck teratomas. We conducted a scoping review to aggregate and report cumulative outcomes of patients who underwent excision surgery. The current literature is limited to isolated case reports and a few case series—mostly due to the rarity of congenital head and neck teratomas. We report the largest combined study to date, with data from 137 patients that focused on surgical management and outcomes.

The obstructive and compressive nature of head and neck teratomas results in a majority of infants suffering from respiratory distress at birth. Patients with anterior cervical and oropharyngeal teratomas commonly present with an airway compromise (66%). Our reported overall respiratory distress rate of 58% is similar to previous studies.^7^, ^17^ Our study had 21 (n = 21/137, 15%) patients who successfully underwent the EXIT procedure to establish an airway. The EXIT was first developed in the 1990s for airway protection as part of congenital diaphragmatic hernia management.^18^ Since then, the indications of the procedure have expanded to other lesions that compromise fetal airways, such as cystic lymphatic malformation, salivary gland cysts, severe micrognathia, fetal goiters, and cervical teratomas.^19^ Maintenance of uteroplacental blood flow allows fetal bronchoscopy, laryngoscopy, endotracheal intubation, tracheostomy, and cannulation for extracorporeal membrane oxygenation.^18^ An interdisciplinary team for fetal airways should include an otolaryngologist, neonatologist, maternal‐fetal specialist, and maternal‐fetal anesthesiologist for optimal execution of this procedure.^20^ Utilizing the EXIT procedure has been shown to significantly reduce perinatal mortality with an overall survival rate of 83%.^21^ A systematic review of 235 cases utilizing EXIT reported congenital teratoma as the most frequent diagnosis (46.4%). This study reported fetal death in 17% of the cases and postpartum hemorrhage (4.5%) as the most common maternal adverse event.^22^ Along with the potential benefits, the care team should discuss mortality and morbidity related to the procedure with the family.

Our study provides robust data for surgeons to understand complications and additional considerations related to head and neck teratoma surgery. The most common postoperative complications were vocal cord paralysis in 4 patients (n = 4/137, 4%) and tracheomalacia in 2 patients (n = 2/137, 2%). Due to the complex distorted anatomy caused by large cervical teratomas, patients may suffer from hypoplastic vocal cords or iatrogenic neural injury.^23^ Patients with these 2 operative complications tended to have large tumors (range: 200‐2160 cc) that were often located in the anterior neck. In addition, the mass can affect the patency of the airway.^23^ The overall surgical complication rates were lower (11%) in this cohort compared to other large congenital obstructive neck masses such as macrocystic lymphatic malformation (17%).^24^

Postoperatively, tracheomalacia or vocal cord paralysis can still lead to a tracheostomy in a subset of patients. Twenty patients in our study required tracheostomy during excision operation, and they were all decannulated with a median of 60 days. Additional surgeries were required in 26 (n = 26/137, 19%) patients. Most of the patients that did require additional surgeries in our study only required 1 additional surgery. Four patients required more than 1 additional surgery. These procedures included residual mass resection in 8 patients and gastrostomy tube insertion in 4 patients due to feeding difficulty. Six patients only required 1 additional surgery to remove residual mass, whereas 2 patients required 2 additional surgeries to remove residual disease. We report a 2% known postoperative mortality rate. One of those 2 patients suffered from aspiration pneumonia following residual mass excision surgery at 8 months. The second death was attributed to intractable lymphangioma with tracheomalacia at 3 months, though It is unclear if lymphangioma was secondary to teratoma. Noting these risks during the presurgical consent may help prepare parents for possible postoperative morbidities. Parental education and giving thorough information have been shown to set expectations leading to higher care satisfaction.^25^

Rare recurrences have been reported in the literature for these tumors, prompting for close observation following surgery.^26^, ^27^ Our study reported a median follow‐up of 24 months with a mean of 41 months. The current follow‐up guidelines from the National Cancer Institute are not specific to head and neck congenital teratomas.^28^ They recommend close observation with physical exams and a periodic evaluation of AFP levels for all mature and immature extragonadal teratomas.^28^ They recommend imaging every 3 months for the first year and every 6 month for the second year for general group of patients with extracranial germ cell tumors.^28^ A large majority of the surgeons in our study opted for periodic imaging as well during follow‐ups (66%); however, only 3 patients with recurrent disease benefited from frequent imaging.^26^, ^29^, ^30^ All 3 were recurrences in infratemporal fossa speculated to be due to incomplete initial excisions.^26^, ^29^, ^30^ A complete head and neck physical exam is crucial during follow‐up visits. Our study demonstrates that a thorough physical exam was essential in detecting recurrences in 50% of the cases (Table 4). This study can serve to provide data for follow‐up guidelines specifically for congenital head and neck teratomas. Based on the information from this scoping review it is reasonable to consider periodic physical exams or ultrasounds as a primary tool for recurrence surveillance for congenital teratomas of the head and neck region that are not involving the skull base. One issue with repeated CT in pediatric patients is that it adds to the increased cost of follow‐up visits, but it also poses risk for malignancies in adulthood due to the increased radiation exposure.^31^ More invasive imaging, such as CT or MRI, can be considered to be reserved for cases with suspected incomplete initial resection, tumor locations not accessible by ultrasound, and/or teratomas extending to the infratemporal fossa.

While we gathered and objectively screened all relevant literature providing sufficient posttreatment outcomes and follow‐up data, this study does have some limitations. Specifically, a scarcity of publications on this rare disease limited our sample size to 108 studies and only 137 cases. This is a rare disease process so with case reports already comprising much of the literature on this topic, researchers may be less motivated to write and publish outcomes with their small number of experiences. There is not a national‐level database containing long‐term outcomes information, so this study provides surgeons with valuable insight related to congenital head and neck teratoma care. Another inherent limitation of this review would be the possibility of missing relevant studies. We utilized 5 databases to mitigate this possibility. This study is also limited by heterogeneity in reported clinical data and the inability to access complete patient records. Also, the case reports and series in the literature on this topic likely do not represent all congenital teratomas. Further studies focusing on the trends in outcomes of this disease by stratifying time periods would be beneficial.

## Conclusion

This scoping review identified, collated, and analyzed isolated case reports and case series of patients with congenital head and neck teratomas. Overall, we found that most fetuses reached term at delivery and that 58% are born with respiratory distress. Respiratory distress was more common in patients with oropharyngeal and anterior cervical tumors (66%). Patients undergoing resection surgery early with a median duration of 4 days and had minimal perioperative complications. Vocal cord paralysis and tracheomalacia were the most common postoperative complications among patients with large anterior cervical teratomas. Long‐term follow‐ups with physical exams effectively identified recurrent teratomas, which occurred in 6% of patients in our study. Repeated invasive imaging should be reserved for cases with incomplete initial resection or infratemporal extension.

## Author Contributions

**Siddharth Patel**: study design, data collection, analysis, manuscript writing; **Ansley J. Kunnath**: data collection, analysis, manuscript editing; **Jean‐Nicolas Gallant**: study design, analysis, manuscript writing, and editing; **Ryan H. Belcher**: study design, analysis, manuscript editing.

## Disclosures

### Competing interests

No conflict to disclose for this study.

### Funding source

Study supported by the Vanderbilt University Medical Scholars Program and NIGMS of the National Institutes of Health under award number T32GM007347.

## Supporting information

Appendix A: Search terminology for Database (Document provides readers with detailed search terminologies of each database).

Appendix B: Study details (Table summarizes all 108 studies and presents various aspects of study design and outcomes).
