# Supplementary material for: Surgical Management and Outcomes of Pediatric Congenital Head and Neck Teratomas: A Scoping Review
Source: OTO Open. 2023 Aug 9;7(3):e66. doi: 10.1002/oto2.66 (PMC10410334; doi:10.1002/oto2.66)
Supplement: Supplementary file 1 — Appendix A: Search terminology for Database (Document provides readers with detailed search terminologies of each database). [file OTO2-7-e66-s001.docx]

**Appendix B**

Search Terminology for Database:

PubMed:

("Teratoma"[Mesh] OR teratoma[tiab] OR teratomas[tiab]) AND ("Head and Neck Neoplasms"[Mesh] OR "Head"[Mesh] OR "Neck"[Mesh] OR “Oral”[Mesh] OR “Nasal”[Mesh] OR “Oropharyngeal”[Mesh] OR “Laryngeal”[Mesh] OR head[tiab] OR neck[tiab] OR cervical[tiab] OR oral[tiab] OR nasal[tiab] OR oropharyngeal[tiab] OR laryngeal[tiab]) AND ("congenital" [Subheading] OR congenital[tiab])

Embase:

(teratoma:ti,ab OR teratomas:ti,ab) AND ('head and neck tumor'/exp OR 'head'/exp OR head:ti,ab OR 'neck'/exp OR neck:ti,ab OR cervical:ti,ab OR laryngeal:ti,ab OR oropharyngeal:ti,ab OR oral:ti,ab OR nasal:ti,ab) AND congenital:ti,ab AND [english]/lim AND [<1966-2021]/py

Web of Science:

TS=(teratoma OR teratomas) AND TS=(head OR neck OR cervical OR laryngeal OR nasal OR oral OR oropharyngeal OR oral) AND TS=(congenital)

Cochrane Central Register of Controlled Trials:

(teratoma OR teratomas) AND (head OR neck OR cervical OR laryngeal OR oropharyngeal OR nasal OR oral) AND congenital

Clinicaltrials.gov

(teratoma OR teratomas) AND (congenital OR (head OR neck OR cervical OR laryngeal OR oropharyngeal OR oral OR nasal))
