# Supplementary material for: Surgical Management and Outcomes of Pediatric Congenital Head and Neck Teratomas: A Scoping Review
Source: OTO Open. 2023 Aug 9;7(3):e66. doi: 10.1002/oto2.66 (PMC10410334; doi:10.1002/oto2.66)
Supplement: Supplementary file 2 — Appendix B: Study details (Table summarizes all 108 studies and presents various aspects of study design and outcomes). [file OTO2-7-e66-s002.docx]

| **First Author** | **Journal Name** | **Study year** | **Country** | **Type of Study** | **Number of Patients with Congenital Cervical Teratoma** | **Recurrent Cases** | **Follow-up range (months)** |
| --- | --- | --- | --- | --- | --- | --- | --- |
| R. Azizkhan | J of Pediatric Surgery | 1905 | United States | Case Series | 1 | 0 | 108 |
| L. Muscatello | Eur Arch Otorhinolaryngology | 1905 | Italy | Case Report | 1 | 1 | 82 |
| A. P. Miller | Cancer | 1966 | United States | Case Report | 1 | 0 | 60 |
| S. B. Aloojee | SA Medical Journal | 1980 | South Africa | Case Report | 1 | 0 | 26 |
| P. E. Rose | J Pediatric Surg | 1982 | United Kingdom | Case Report | 1 | 0 | 18 |
| G. A. Antoine | Laryngoscope | 1985 | United States | Case Report | 1 | 0 | 52 |
| T. E. Wiswell | J Pediatric Surg | 1986 | USA | Case Report | 1 | 0 | 8 |
| A. D. Alter | J Pediatric Surg | 1987 | United States | Case Report | 1 | 0 | 52 |
| L. D. Holinger | Ann Otol Rhinol Laryngol | 1987 | United States | Case Report | 1 | 0 | 24 |
| Y. P. Talmi | J Laryngology Otol | 1988 | Israel | Case Report | 1 | 0 | 4 |
| R. W. Byard | J Pediatric Child Health | 1989 | Canada | Case Series | 6 | 1/6 | 24-60 |
| L. P. Dehner | Hum Pathology | 1989 | United States | Case Series | 2 | 0/2 | 13-36 |
| K. Maeda | J Pediatric Surg | 1989 | Japan | Case Report | 1 | 0 | 5 |
| T. Touran | J of Pediatric Surgery | 1989 | United States | Case Report | 1 | 0 | 12 |
| A. J. Jawad | Z Kinderchir | 1990 | Saudi Arabia | Case Report | 1 | 0 | 6 |
| M. F. Kelly | Ann Otol Rhinol Laryngol | 1990 | United States | Case Report | 1 | 0 | 14 |
| P. J. Catalano | Arch Otolaryngology Head Neck Surg | 1991 | United States | Case Report | 1 | 0 | 12 |
| G. Katona | Int J Pediatric Otorhinolaryngology | 1991 | Hungary | Case Report | 1 | 0 | 6 |
| A. K. Lalwani | Int J Pediatric Otorhinolaryngology | 1991 | United States | Case Report | 1 | 0 | 18 |
| L. P. Rybak | Arch Otolaryngology Head Neck Surg | 1991 | Canada | Case Series | 1 | 0 | 120 |
| Y. el-Sayed | J Laryngology Oto | 1992 | Saudi Arabia | Case Report | 1 | 0 | 24 |
| R. M. Conran | Am J Perinatal | 1993 | United States | Case Report | 1 | 0 | 48 |
| A. S. Jaarsma | Eur J Pediatric | 1993 | Netherlands | Case Report | 1 | 0 | 108 |
| M. A. Rothschild | Arch Otolaryngology Head Neck Surg | 1994 | United States | Case Report | 1 | 0 | 12 |
| F. Biglioli | Int J Oral Maxillofacial Surg | 1995 | Italy | Case Report | 1 | 0 | 18 |
| N. M. Cunchillos | J Laryngology Otol | 1996 | Spain | Case Report | 1 | 0 | 6 |
| S. K. Chowdhary | Pediatric Surg Int | 1997 | South Africa | Case Report | 1 | 0 | 9 |
| K. Uchida | Pediatric Surg Int | 1997 | Japan | Case Report | 1 | 0 | 24 |
| M. M. April | Laryngoscope | 1998 | United States | Case Series | 3 | 0/3 | 52-128 |
| F. Elmasalme | Eur J Pediatric Surg | 1998 | Canada | Case Series | 6 | 0/6 | 15-96 |
| G. Lanzino | Neurosurgery | 1998 | United States | Case Report | 1 | 0 | 48 |
| A. G. Oliveira-Filho | J Pediatric Surg | 1998 | Brazil | Case Report | 1 | 0 | 12 |
| I. G. Coppit | Int J of Pediatric Otorhinolaryngology | 2000 | United States | Case Report | 1 | 1 | 5 |
| R. Jarrahy | J Craniofacial Surg | 2000 | United States | Case Report | 1 | 0 | 108 |
| T. Morita | Am J Otolaryngology | 2000 | Japan | Case Report | 1 | 0 | 36 |
| S. C. Shetty | Ear Nose Throat J | 2000 | India | Case Report | 1 | 0 | 14 |
| R. Carrasco | Clin Pediatric (Phila) | 2001 | Spain | Case Report | 1 | 0 | 24 |
| C. C. Liang | Ann Plastic Surg | 2002 | Taiwan | Case Report | 1 | 0 | 12 |
| J. Y. Sichel | Int J Pediatric Otorhinolaryngology | 2002 | Israel | Case Report | 1 | 0 | 48 |
| J. H. Yoon | Oral Surg Oral Med Oral Pathology Oral Radiology | 2002 | Korea | Case Report | 1 | n/a | 0 |
| S. J. Bergé | Br J Oral Maxillofacial Surg | 2003 | Germany | Case Report | 1 | 0 | 36 |
| K. Haghighi | J Oral Maxillofacial Surg | 2003 | United States | Case Report | 1 | 0 | 12 |
| R. Mladina | J Craniofacial Surg | 2003 | Croatia | Case Report | 1 | 0 | 48 |
| W. A. Tjalma | J Pediatric Surg | 2003 | Belgium | Case Report | 1 | 1 | 12 |
| S. Becker | Br J Oral Maxillofacial Surg | 2005 | Germany | Case Report | 1 | 1 | 78 |
| T. Noguchi | Oral Surg Oral Med Oral Pathology Oral Radiology | 2005 | Japan | Case Report | 1 | 0 | 60 |
| G. K. Parvathidevi | Indian J Pediatric | 2005 | India | Case Report | 1 | 0 | 24 |
| C. Sader | Auris Nasus Larynx | 2005 | Australia | Case Report | 1 | 0 | 18 |
| T. Tamura | Asian J Surg | 2005 | Japan | Case Report | 1 | 0 | 60 |
| Z. Ulger | Turk J Pediatric | 2005 | Turkey | Case Report | 1 | 0 | 24 |
| M. Dadmehr | J Neurosurgery | 2006 | Iran | Case Report | 1 | 0 | 6 |
| R. E. Benson | Br J Oral Maxillofacial Surg | 2007 | United Kingdom | Case Report | 1 | 0 | 24 |
| H. H. Chiu | J Formos Med Assoc | 2007 | Taiwan | Case Series | 2 | 0/2 | 3-8 |
| S. F. Rda | Br J Oral Maxillofacial Surg | 2007 | Brazil | Case Report | 1 | 0 | 12 |
| A. Hossein | Am J Otolaryngology | 2007 | Iran | Case Report | 1 | 0 | 18 |
| A. Sayan | J Perinat Med | 2007 | Turkey | Case Series | 2 | 0/2 | 60 |
| N. Choudhury | Indian J Pediatric | 2008 | India | Case Report | 1 | 0 | 6 |
| M. E. Huth | J Pediatric Surg | 2008 | Switzerland | Case Report | 1 | 0 | 12 |
| B. Kumar | J Pediatric Surg | 2008 | India | Case Report | 1 | 0 | 12 |
| F. M. Makki | Eur Arch Otorhinolaryngology | 2008 | Saudi Arabia | Case Report | 1 | 0 | 6 |
| R. W. Byard | Pediatric Pathology | 2009 | Australia | Case Report | 1 | n/a | 54 |
| F. J. Halbertsma | Acta Pediatric, International Journal of Pediatrics | 2009 | Netherlands | Case Report | 1 | 0 | 12 |
| J. He | Oral Surg Oral Med Oral Pathology Oral Radiology | 2009 | Shanghai | Case Series | 2 | 0/2 | 10-24 |
| A. E. Rodin | Pediatric Pathology | 2009 | United States | Case Report | 1 | 0 | 12 |
| B. Y. Wong | Int J Pediatric Otorhinolaryngology | 2009 | China | Case Series | 4 | 0 | 10 |
| N. N. Andrade | British J of Oral and Maxillofacial Surgery | 2010 | India | Case Report | 1 | 0 | 18 |
| B. Bianchi | J of Oral and Maxillofacial Surgery | 2010 | Italy | Case Report | 1 | 0 | 9 |
| N. Isik | Childs Nervous Syst | 2010 | Turkey | Case Report | 1 | 0 | 6 |
| A. Mirshemirani | Iranian Journal of Pediatrics | 2010 | Iran | Case Report | 1 | 0 | 72 |
| S. Sumiyoshi | Int J Oral Maxillofacial Surg | 2010 | Japan | Case Report | 1 | 0 | 48 |
| A. Ismail | Egyptian Journal of Radiology and Nuclear Medicine | 2011 | Nigeria | Case Report | 1 | 0 | 48 |
| L. E. Al-Khurrhi | J. of Craniofacial Surgery | 2012 | Iraq | Case Report | 1 | 0 | 18 |
| M. Bahgat | BMJ Case Rep | 2012 | Egypt | Case Report | 1 | 0 | 26 |
| O. Bayir | Turk Pediatric Arsivi | 2012 | Turkey | Case Report | 1 | 0 | 80 |
| C. Bonet | J Oral Maxillofacial Surg | 2012 | Spain | Case Series | 5 | 0/5 | 36-108 |
| M. Ibrahimov | J Craniofacial Surg | 2012 | United States | Case Report | 1 | 0 | 36 |
| K. Kamrani | Iranian Journal of Pediatrics | 2012 | Iran | Case Report | 1 | 0 | 12 |
| A. Ozturk | Fetal Pediatric Pathology | 2012 | Turkey | Case Report | 1 | 0 | 10 |
| V. Rangachari | J Laryngology Otol | 2012 | India | Case Report | 1 | 0 | 4 |
| G. Barthod | J Obstetric Gynecology | 2013 | France | Case Series | 2 | 0/2 | 36-364 |
| S. Dakpé | Int J Oral Maxillofacial Surg | 2013 | France | Case Report | 1 | 0 | 36 |
| A. M. Fink | J of Pediatric Neuroradiology | 2013 | Australia | Case Report | 1 | 0 | 108 |
| P. Laje | J Pediatric Surg | 2013 | United States | Case Report | 1 | 0 | 72 |
| M. Manchali | J. of Clinical Imaging Science | 2014 | India | Case Report | 1 | 0 | 189 |
| A. Aubin | Euro Ann Otorhinolaryngology Head Neck Dis | 2014 | France | Case Series | 4 | 2/4 | 52-104 |
| J. Courtier | Pediatric Radiology | 2014 | Mexico | Case Report | 1 | 0 | 3 |
| H. O. Gezer | Pediatrics and Neonatology | 2014 | Turkey | Case Series | 2 | 0/2 | 12-May |
| H. H. Han | J Craniofacial Surg | 2014 | South Korea | Case Report | 1 | 0 | 12 |
| E. Moreddu | Int J Pediatric Otorhinolaryngology | 2015 | France | Case Report | 1 | 0 | 18 |
| X. Pang | J Craniofacial Surg | 2015 | United States | Case Report | 1 | 1 | 11 |
| A. Posod | BMC Pediatric | 2015 | Austria | Case Report | 1 | 0 | 9 |
| K. J. Shetty | J Cancer Res Ther | 2015 | India | Case Report | 1 | 0 | 12 |
| J. Jang | Journal of Pediatric Surgery Case Reports | 2016 | South Korea | Case Report | 1 | 0 | 12 |
| S. Ulrika | Neuro-Oncology | 2016 | Sweden | Case Report | 1 | 0 | 21 |
| P. N. Dang | Br J Oral Maxillofacial Surg | 2017 | France | Case Report | 1 | 0 | 12 |
| S. Al-Shaqsi | Archives of Plastic Surgery | 2018 | Canada | Case Report | 1 | 0 | 26 |
| M. Gaffuri | J Pediatric Surg | 2018 | Italy | Case Series | 2 | 1/2 | 6-108 |
| H. T. Thong | Rawal Medical Journal | 2018 | Malaysia | Case Report | 1 | 0 | 6 |
| R. S. Tunes | Craniomaxillofacial Trauma and Reconstruction | 2018 | Brazil | Case Report | 1 | 0 | 4 |
| K. Beckers | Int J Pediatric Otorhinolaryngology | 2019 | Belgium | Case Series | 3 | 0/3 | 93-214 |
| E. O. Ezzi | Oral and Maxillofacial Surgery Cases | 2019 | Switzerland | Case Report | 1 | 0 | 52 |
| O. Hochwald | J Med Case Rep | 2019 | Israel | Case Report | 1 | 0 | 3 |
| K. Masahata | Pediatric Surg Int | 2019 | Japan | Case Series | 2 | 0/2 | 8-168 |
| W. Tigabie | J of Pediatric Surgery Case Reports | 2020 | Ethiopia | Case Report | 1 | 0 | 3 |
| E. Yhoshu | Afr J Pediatric Surg | 2020 | India | Case Report | 1 | 0 | 21 |
| K. Quinn | Journal of Pediatric Surgery Case Reports | 2021 | United States | Case Report | 1 | 0 | 16 |
| P. Zhu | J Int Med Res | 2021 | China | Case Series | 2 | 0 | 12 |

**Supplemental: Appendix A: Study Details**
